# A Wearable Channel Selection-Based Brain-Computer Interface for Motor Imagery Detection

**DOI:** 10.3390/s16020213

**Published:** 2016-02-06

**Authors:** Chi-Chun Lo, Tsung-Yi Chien, Yu-Chun Chen, Shang-Ho Tsai, Wai-Chi Fang, Bor-Shyh Lin

**Affiliations:** 1 Institute of Electrical and Control Engineering, National Chiao Tung University, Hsinchu 300, Taiwan; chichun86@gmail.com (C.-C.L.); shanghot@mail.nctu.edu.tw (S.-H.T.); 2 Department of Engineering and Maintenance, Chang Gung Memorial Hospital, Kaohsiung 833, Taiwan; g101@cgmh.org.tw; 3 Institute of Imaging and Biomedical Photonics, National Chiao Tung University, Tainan 711, Taiwan; e01052788@hotmail.com; 4 Department of Electronics Egineering, National Chiao Tung University, Hsinchu 300, Taiwan; wfang@mail.nctu.edu.tw; 5 Department of Medical Research, Chi-Mei Medical Center, Tainan 710, Taiwan

**Keywords:** brain-computer interface, motor imagery, channel selection, spatial filter

## Abstract

Motor imagery-based brain-computer interface (BCI) is a communication interface between an external machine and the brain. Many kinds of spatial filters are used in BCIs to enhance the electroencephalography (EEG) features related to motor imagery. The approach of channel selection, developed to reserve meaningful EEG channels, is also an important technique for the development of BCIs. However, current BCI systems require a conventional EEG machine and EEG electrodes with conductive gel to acquire multi-channel EEG signals and then transmit these EEG signals to the back-end computer to perform the approach of channel selection. This reduces the convenience of use in daily life and increases the limitations of BCI applications. In order to improve the above issues, a novel wearable channel selection-based brain-computer interface is proposed. Here, retractable comb-shaped active dry electrodes are designed to measure the EEG signals on a hairy site, without conductive gel. By the design of analog CAR spatial filters and the firmware of EEG acquisition module, the function of spatial filters could be performed without any calculation, and channel selection could be performed in the front-end device to improve the practicability of detecting motor imagery in the wearable EEG device directly or in commercial mobile phones or tablets, which may have relatively low system specifications. Finally, the performance of the proposed BCI is investigated, and the experimental results show that the proposed system is a good wearable BCI system prototype.

## 1. Introduction

The motor imagery-based brain-computer interface (MI-based BCI) is the direct communication pathway between an external machine and the brain so that the user can control the external machine by the mental activity of motor imagery [1,2]. MI-based BCI is a type of active BCI which does not require any external stimuli [3]. The event related to motor imagery will result in an amplitude decrease or increase of EEG oscillatory components generated from the sensorimotor cortex, and they are also called event-related desynchronization (ERD) and event-related synchronization (ERS), respectively [4]. Therefore, the design of MI-based BCIs is based on the above phenomena reflecting the sensorimotor brain activity. Different from other BCIs, such as P300-based BCIs and steady-state visual evoked potential (SSVEP) BCIs [5,6], the operation of an MI-based BCI does not require any external visual stimuli, and therefore, the applications of an MI-based BCI are more convenient and practicable.

In order to improve the accuracy of MI-based BCIs, several spatial filters, such as common spatial pattern (CSP), independent component analysis (ICA), Laplacian derivations, and common average reference (CAR) [7,8,9], are proposed to enhance the EEG features related to motor imagery in the specific region. One of the alternative approaches is channel selection. Channel selection is a kind of feature selection, which can be characterized as a special filter [10] used for selecting related-feature channels and removing irrelevant or noisy channels to improve the BCI performance [11]. Lan *et al.*, proposed an EEG channel selection approach using the mutual information technique [12]. The mutual information between the channels and the class labels was used for ranking all the channels. Arvaneh *et al.*, proposed the sparse common spatial pattern (SCSP) algorithm to remove noisy or irrelevant channels without affecting the classification accuracy [13]. Schröder *et al.*, proposed the recursive channel elimination (RCE) method, which could disregard task-irrelevant channels [10]. Lal *et al.*, proposed feature selection algorithms including zero-norm optimization (10-Opt) and recursive feature elimination (RFE) which were based on the training of support vector machines (SVM) [11]. Although the approach of channel selection can provide the advantages of removing irrelevant channels or selecting few meaningful EEG features to improve the BCI performance, multi-channel EEG signals have to be acquired and transmitted to the back-end computer to perform the approach of channel selection. Moreover, the conventional bulk EEG machine and lots of EEG electrodes with conductive gel have to be used for acquiring multi-channel EEG signals. The above issues will limit the BCI applications and cause inconveniences in daily life use.

In this study, a novel wearable channel selection-based brain-computer interface is proposed for motor imagery detection. The proposed system contains a front-end wearable EEG device and a back-end host system. Here, retractable comb-shaped active dry electrodes are proposed and used in the wearable EEG device to measure the EEG signals on hairy sites without conductive gel. In the wearable EEG device, an analog circuit of CAR spatial filter is also designed to perform a spatial filter function without any computational complexity. Moreover, the channel selection function is built in the firmware of the wearable EEG device. Different from other channel selection-based BCIs, the channel selection function could be performed in the front-end wearable EEG device directly by using the analog CAR spatial filter and the channel selection firmware, and only the EEG signals in selected channels are transmitted to the back-end host system. This also improves the practicability of detecting motor imagery in the wearable EEG device directly or in commercial mobile phones or tablets, which may have relatively low computing power and memory, to reduce the limitations of BCI applications. In the future, the function of detecting motor imagery could also be implemented in the firmware of wearable EEG devices to control the external devices directly. Therefore, the proposed system is a good system prototype, and being applied to different BCI applications in daily life will become more practicable.

## 2. Methods

### 2.1. System Hardware Design and Implementation

**Figure 1:**
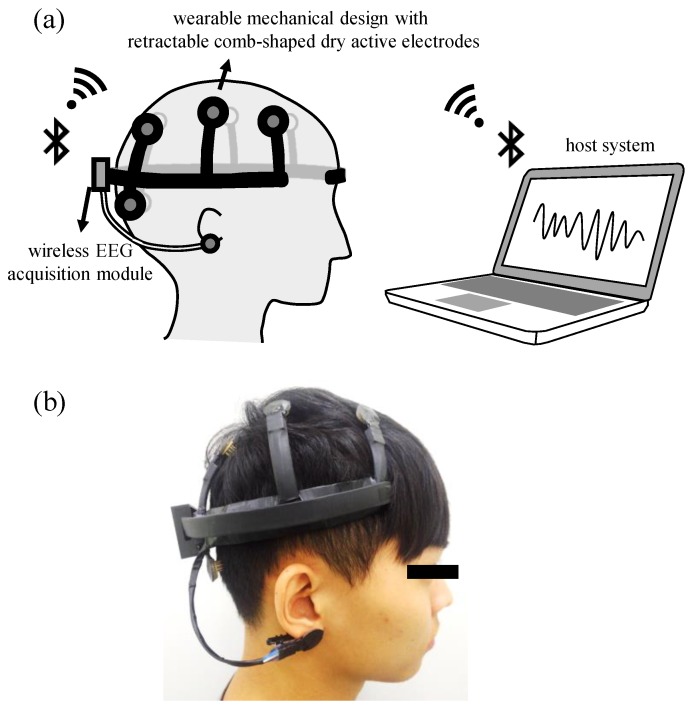
Basic scheme (**a**) and photograph (**b**) of proposed wearable motor-imagery brain-computer interface.

The proposed system contains a front-end wearable EEG device and the back-end host system, and its basic scheme and a photograph are shown in Figure 1a,b, respectively. Here, the retractable comb-shaped dry active electrodes built in the front-end wearable EEG device are designed to measure the EEG signals on a hairy site without conductive gel. By using the property of the proposed active electrodes for measuring EEG without conductive gel, the design of the wearable EEG device becomes practicable and offers the advantages of easy wear and easy use. The EEG signals, acquired by the proposed active electrodes, would be amplified and filtered by the wireless EEG acquisition module built in the front-end wearable EEG device, and then the module would perform the function of channel selection to collect the meaningful channels. Next, the EEG signals of the meaningful channels would be transmitted to the back-end host system wirelessly via Bluetooth. Finally, the back-end host system could detect the event of motor imagery and send control commands to the external machine.

#### 2.1.1. Front-End Wearable EEG Device

**Figure 2:**
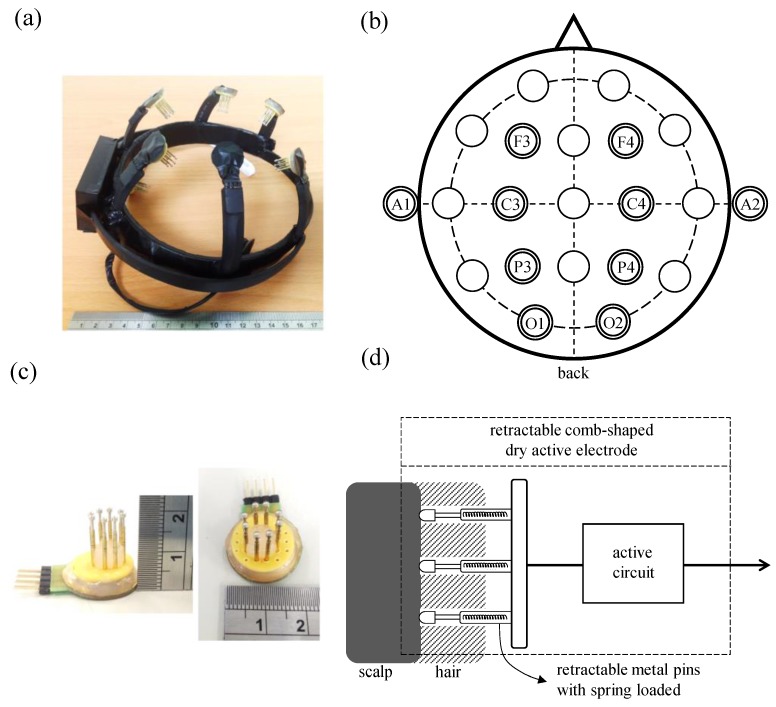
(**a**) Structure of the wearable mechanical design; and (**b**) locations; (**c**) photograph and (**d**) basic scheme of retractable comb-shaped dry active electrodes.

The front-end wearable EEG device mainly consists of eight retractable comb-shaped dry active electrodes, a wireless EEG acquisition module, and a wearable mechanical design. Here, the structure of the wearable mechanical design is illustrated in Figure 2a. The wearable mechanical design is made of flexible plastic, and its structure contains a circle structure part and a claw-shaped structure part. The circle structure part is designed to fix the wearable mechanical design on the head, and the flexibility properties of the flexible plastic allow the circle structure to fit different head sizes. The retractable comb-shaped dry active electrodes are embedded in these claw-shaped structures, and the flexibility properties of these claw-shaped structures provide a suitable pressure on these active electrodes to allow them to contact with the skin well. According to the international 10–20 EEG system, these retractable comb-shaped dry active electrodes are placed at the locations F3, C3, P3, O1, F4, C4, P4, and O2, and the system grounding electrode and the reference electrode are placed at A1 and A2, respectively. The placement of the active electrodes is shown in Figure 2b.

A photograph of the proposed retractable comb-shaped dry active electrodes is shown in Figure 2c. The diameter of the electrode base is 2 cm and its height is about 2 cm. Each electrode contains eight retractable spring-loaded metal pins, which are coated with Au, as illustrated in Figure 2d. By using a retractable metal pin structure, it can separate the hair layer and provide a suitable pressure to maintain a good skin-electrode contact condition to acquire good-quality EEG signals. Moreover, the active circuit in the proposed electrodes could provide an ultra-high input impedance to reduce the influence of the high skin-electrode interface impedance and decrease the attenuation of EEG signals [14]. Therefore, by using the proposed retractable comb-shaped dry active electrodes, EEG signals could be acquired without conductive gel.

**Figure 3:**
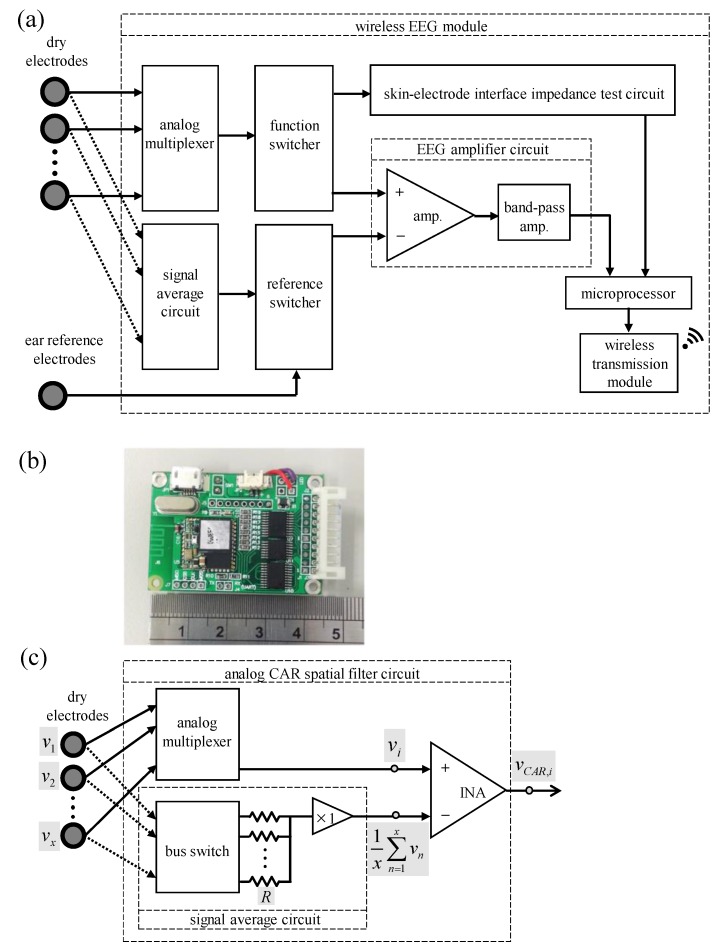
Block diagram (**a**,**b**) photograph of proposed wireless EEG acquisition module; and (**c**) basic schematic of analog CAR spatial filter circuit.

The block diagram and a photograph of the wireless EEG acquisition module are shown in Figure 3a,b, respectively. The size of the proposed module is about 45 × 30 × 5 mm^3^, and it mainly contains several parts, including an analog multiplexer, an analog CAR filter circuit, a functional switcher, a reference switcher, an EEG amplifier circuit, a skin-electrode interface impedance test circuit, a microprocessor, and a wireless transmission module. Here, the analog 8 to 1 multiplexer (SN74LV4051A, Texas Instruments, Dallas, TX, USA) is used for deciding the acquired EEG channels from the eight EEG channels. The switching frequency of the analog multiplexer is set to 2000 Hz. The functional switcher (SN74CBTLV3126, Texas Instruments) is designed to switch the acquired EEG channel to the EEG amplifier circuit or the skin-electrode interface impedance test circuit. Here, the skin-electrode interface impedance test circuit provides a 10 Hz sine wave which is used for passing through the skin to evaluate the skin-electrode interface impedance [15]. The EEG amplifier circuit contains an instrumentation amplifier and a band-pass amplifier (AD8609, Analog Device, Norwood, MA, USA), and its total gain is set to 5000 times with the frequency band of 5–30 Hz. The proposed system first performs the skin-electrode interface impedance test circuit to check the skin-electrode contact condition and then performs the EEG amplifier circuit to acquire EEG signals continuously. Here, the reference switcher is designed to select the raw EEG signals obtained from the reference electrode or the EEG signals filtered by the analog CAR filter circuit as the reference signal of the EEG amplifier circuit. The analog CAR spatial filter circuit is designed to enhance the EEG features related to motor imagery in the specific region [8], and its schematic circuit is illustrated in Figure 3c. Let *x* denote the total number of the EEG channels, and let *v_i_* denote the i-th channel EEG signals. When the bus switch opens, each EEG channel would connect in series with the resistance *R*. Because the values of the connected resistances are the same, this can be viewed as a signal average circuit. If the output signal of the signal average circuit is selected as the reference signal of the EEG amplifier circuit by the reference switcher, the output signal $v_{CAR,i}$ of the i-th EEG channel filtered by the analog CAR filter could be expressed by:

(1) $$v_{CAR,i}=\left(v_{i}-\frac{1}{x}\sum_{n=1}^{x}v_{n}\right)\times gain_{EEG\;Amplifier}$$

where $gain_{EEG\;Amplifier}$ denotes the total gain of the EEG amplifier circuit.

After being amplified by the EEG amplifier circuit, the acquired EEG signals would be digitized by a 12-bit analog-to-digital converter (ADC) built in the microprocessor (MSP430, Texas Instruments) with a sampling rate of 250 Hz. Next, the firmware of the microprocessor performs the function of channel selection, and the EEG signals of the selected channels are sent to the wireless transmission circuit to be transmitted to the back-end host system wirelessly via Bluetooth. Here, the wireless transmission circuit is compatible with the Bluetooth v2.0+EDR specifications.

#### 2.1.2. Back-End Host System

A commercial tablet with the operation system of Windows 8 is used as the platform of the back-end host system. A BCI monitoring program built in the back-end host system is developed in Microsoft C# to provide the functions of monitoring EEG signals, skin-electrode interface impedance testing, channel selection, and detecting the motor imagery event.

### 2.2. System Software Design

An event of hand motion or hand motor imagery results in the change of EEG power in the sensorimotor cortex and reflects on the mu rhthm (8~13 Hz) and beta rhythm (13~30 Hz). This change of EEG power is called related desynchronization (ERD) and event-related synchronization (ERS) [16,17]. The occurrence of a hand motion or motor imagery results in a decrease of the EEG power in mu rhythm generated from the sensory-motor cortex, and the end of the hand motion or motor imagery causes an increase of EEG power in the mu and beta rhythms. In this study, the development of the channel selection algorithm and the motor imagery detection algorithm are based on the above phenomena. The channel selection algorithm would choose the two channels that contain the most obvious motor imagery features for the left hand motor imagery and the right hand motor imagery, respectively. The motor imagery detection algorithm compares the ERD of these two selected channels and can successfully distinguish between the left or the right hand motor imagery.

#### 2.2.1. Channel Selection Algorithm

**Figure 4:**
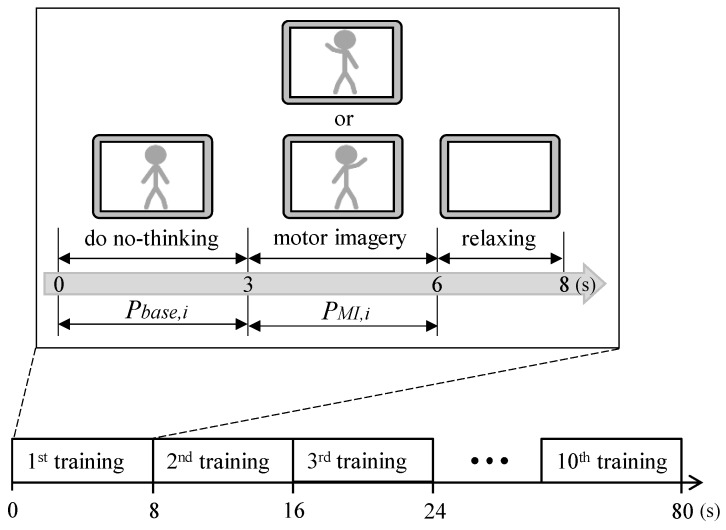
Illustration of the procedure of channel selection algorithm.

In the channel selection function, the proposed BCI monitoring program would periodically give a cue to instruct a user to perform motor imagery (no thinking, the left hand motor imagery, or the right hand motor imagery), and the firmware of the wireless EEG acquisition module would select the channels that provide the most obvious ERD features related with the motor imagery. The procedure of the channel selection algorithm is shown in Figure 4. In the first 3 s of each training cycle, the program presents the cue that shows a gray matchstick man in a static state to instruct the user to do no-thinking. During the next 3 s, the program would randomly present the cue that shows the gray matchstick man under the raising left or right hand to instruct the user to perform left or right motor imagery three times continuously. During the last 2 s, the program shows nothing to instruct the user to relax for a while. The period of each training cycle is 8 s, and the whole process of channel selection would perform 10 testing cycles.

In each training cycle, the firmware of the wireless EEG acquisition module calculates the ERD quantification of each channel simultaneously. The acquired EEG signals would first be passed through a band-pass digital filter (biquad tweaked Butterworth type filter) with the frequency band 8~13 Hz to reserve EEG features related to motor imagery. Before the user is instructed to perform motor imagery, the average EEG power during the first 3 s of each training cycle is calculated as the EEG power baseline. Next, the average EEG power between 3 s and 6 s of each training cycle is calculated as the EEG power of motor imagery. Then, the i-th channel ERD quantification *ERD_i_* [4,18] could be calculated by:

(2) $$ERD_{i}=\frac{P_{MI,i}-P_{base,i}}{P_{base,i}}\times 100\%$$

where $P_{MI,i}$ and $P_{base,i}$ denote the i-th channel EEG power of motor imagery and the EEG power baseline, respectively. After the above channel selection algorithm procedure, the channel which provides the smallest averaged *ERD_i_* for left or right motor imagery, would be selected as the meaningful channel, and they are presented as $Channel_{L}$ and $Channel_{R}$, respectively. For the function of motor imagery detection, only the EEG signals in $Channel_{L}$ and $Channel_{R}$ would be transmitted to the back-end host system.

#### 2.2.2. Motor Imagery Detection Algorithm

The procedure of the motor imagery detection algorithm is similar to the channel selection algorithm. Here, the proposed BCI monitoring program would also periodically give a cue to instruct the user to perform motor imagery, and the wireless EEG acquisition module would calculate and send the ERD quantification of the selected channel to the back-end host system to estimate the motor imagery event. In the first 3 s of each cycle, the program presents the cue that shows a gray matchstick man in a static state to instruct the user to do no-thinking. During the next 3 s, the program would randomly present the cue that shows the gray matchstick man under raising both of the left and the right hands to instruct the user to perform motor imagery 3 times continuously. Next, during the last 2 s, the user is instructed to relax. After completing each motor imagery test, ERD quantifications of each selected channel would be calculated by Equation (2). If the ERD quantification of *Channel_L_*/ *Channel_R_* is smaller than the given threshold, and the minimum, the mental event could be viewed as the event of right/left hand motor imagery. If both of the ERD quantifications, *Channel_L_* and *Channel_R_*, are larger than the given threshold, the mental event could be viewed as the idle state. In our experimental results, the average ERD quantification for the idle state is about 1.4% ± 6.4%. Therefore, the threshold of the ERD quantification is defined as −5% in this study. Finally, the mental events of left-hand motor imagery, right-hand motor imagery, and idle state could be detected by comparing the ERD quantification of *Channel_L_* and *Channel_R_*.

## 3. Results

### 3.1. Performance of Retractable Comb-Shaped Dry Active Electrode

In this section, the EEG signal quality measured by the proposed retractable comb-shaped dry active electrodes is investigated. The traditional Ag/AgCl electrode with conductive gel (wet electrode) is considered generally as a standard electrode to measure EEG. Therefore, the EEG signal quality of the proposed retractable comb-shaped dry active electrodes is compared with that of the wet electrodes.

**Figure 5:**
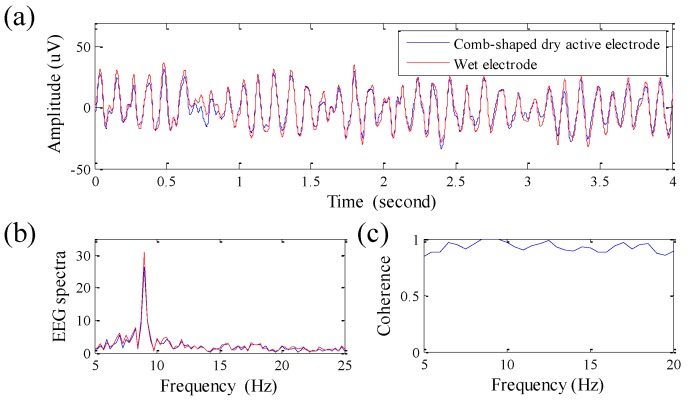
(**a**) EEG signals and (**b**) EEG spectra for the experiment of alpha rhythm; and (**c**) their coherence corresponding to different frequencies.

First, an alpha rhythm experiment is performed. In this experiment, two different types of electrodes are placed at Oz, and the participant is instructed to close his/her eyes to generate alpha rhythms. Here, two different types of electrodes are placed as close to each other as possible. Figure 5a–c show the comparisons of EEG signals and EEG spectra, and their coherence analysis, respectively. The result shows that the EEG signal quality obtained by the retractable comb-shaped dry active electrodes is good and similar to that provided by the wet electrodes, and the alpha rhythm features are clearly presented in the EEG spectra. The correlation between EEG signals obtained by two different types of electrodes is about 95.7%, and their coherence is also high and stable.

**Figure 6:**
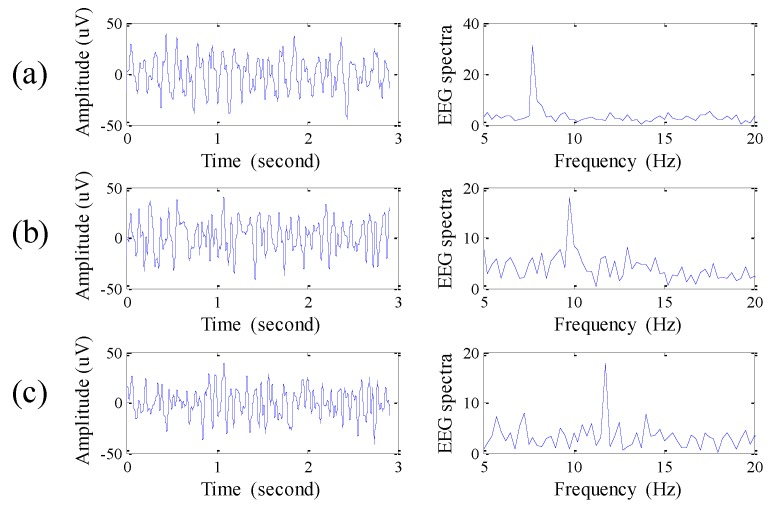
EEG signals and EEG spectra for SSVEP experiment with (**a**) 8 Hz; (**b**) 10 Hz; and (**c**) 12 Hz flashing light sources.

Next, a steady state visually evoked potential (SSVEP) experiment is tested. When the participant watches an external visual stimulus with a specific flashing frequency, EEG signals corresponding to the specific frequency could be observed in the location of Oz. The above phenomenon is called steady state visually evoked potential [19]. Here, a white-light light-emitting diode (LED) module with flashing frequencies of 8, 10 and 12 Hz is used as the source of the external visual stimulus, and the retractable comb-shaped dry active electrodes are placed at Oz. Figure 6 shows the EEG signals and the EEG spectra corresponding to different flashing frequencies. The result shows that the peaks in the EEG spectra accurately reflect the specific flashing frequency.

**Figure 7:**
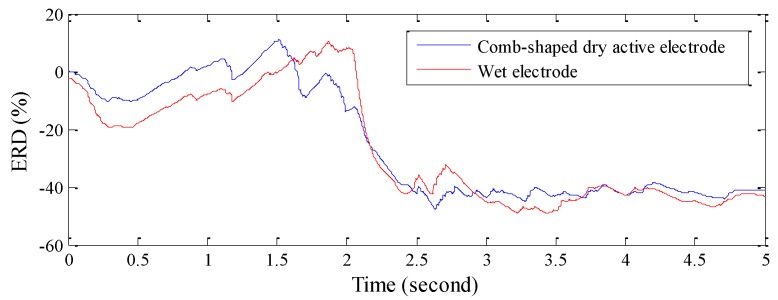
Comparison of ERD quantifications recorded at C3 by using retractable comb-shape dry active electrodes and wet electrodes for right hand motor imagery.

Next, a motor imagery experiment is performed. Here, two different types of electrodes are placed at the C3 location. In the first 2 s, the participant is instructed to do nothing, and the average power of the first 2 s EEG is calculated as the EEG power baseline. Next, the participant is instructed to do right hand motor imagery, and the average power of EEG in mu rhythm is calculated as the EEG power of motor imagery. The change of ERD quantifications with time for different electrodes is shown in Figure 7. After completing the motor imagery, the ERD quantifications of the proposed electrodes and the wet electrodes are −40% and −39%, respectively, and the correlation between ERD quantifications obtained by the two electrodes is 94.2%.

### 3.2. Performance of Channel Selection Function

**Figure 8:**
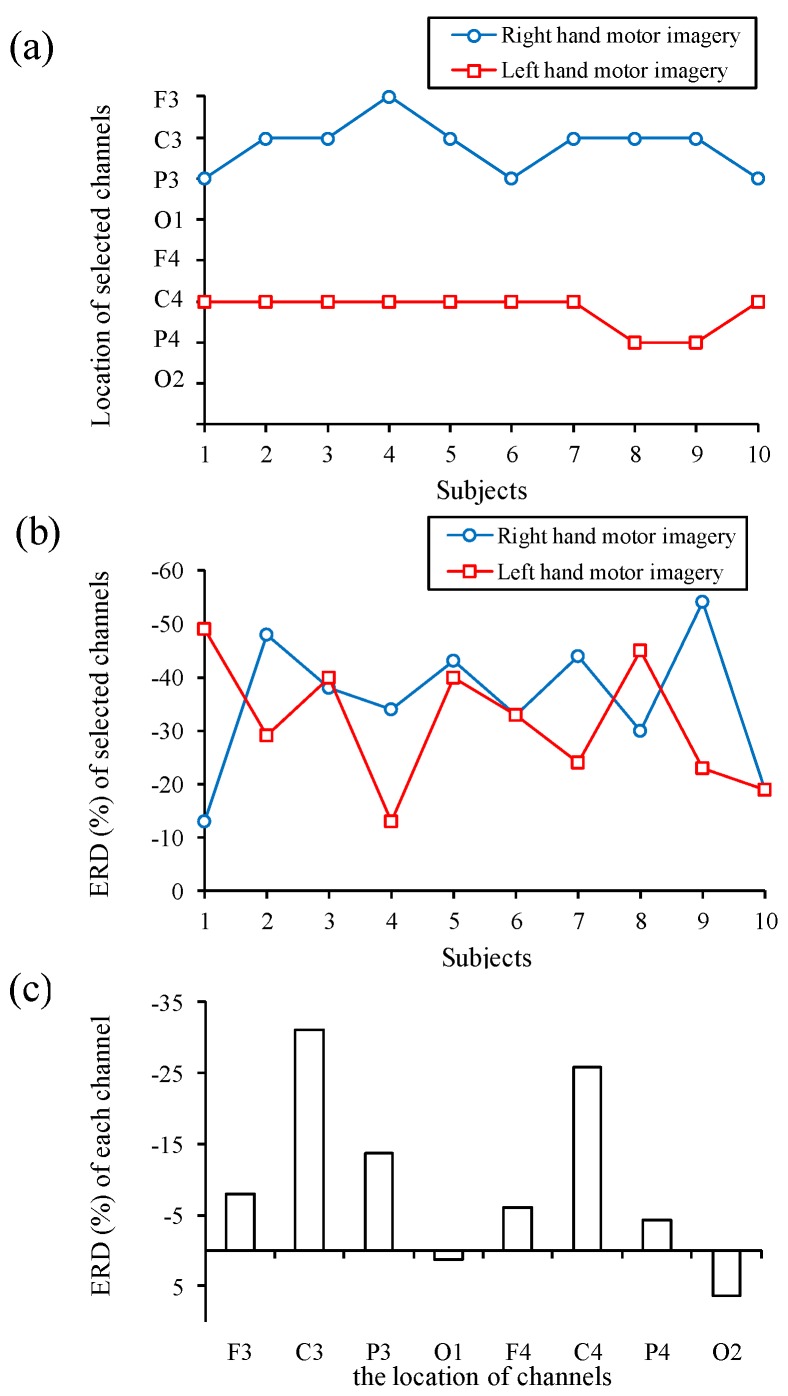
Experimental results for (**a**) locations and (**b**) ERD quantifications of selected EEG channels; and (**c**) averaged ERD quantifications of each EEG channel for the right and the left hand motor imagery.

In this section, the channel selection function performance is investigated. A total of 10 participants, who do not have any experience in operating motor imagery BCIs, performed this experiment. Before this experiment, all participants are trained to operate the proposed system for half an hour and then instructed to take a rest for another half an hour. The experimental results for the location and ERD quantifications of selected channels for different motor imagery are shown in Figure 8a,b, respectively. Most of the selected channel locations for the right and the left hand motor imagery are C3 and C4, respectively. The averaged ERD quantifications of selected channels for the right and the left hand motor imagery are −31.0% ± 19.0% and−25.8% ± 15.2%, respectively. Figure 8c shows the averaged ERD quantifications of each EEG channel corresponding to the right and the left motor imagery. The locations of C3, corresponding to the right hand motor imagery, and C4, corresponding to the left hand motor imagery, provide the obvious ERD features.

### 3.3. Performance of Motor Imagery Detection

In this section, the motor imagery detection performance is investigated. A total of 10 participants performed this experiment. Before the experiment, each participant is instructed to perform the channel selection training, and then the selected channels would be used for detecting the motor imagery. During the experiment of motor imagery detection, the participants are instructed to perform motor imagery15 times, including five times left hand and five times right hand motor imagery and five times idle state. Here, these cues of motor imagery are randomly presented. In order to evaluate the performance of motor imagery detection, several parameters for the classification test are first defined and listed as follows. True positive (*tp*) denotes that the event of right/left hand motor imagery is correctly recognized as right/left hand motor imagery. False positive (*fp*) denotes that the event of idle state is wrongly recognized as right or left hand motor imagery. True negative (*tn*) denotes that the event of idle state is correctly recognized as idle state. False negative (*fn*) denotes that the event of right/left hand motor imagery is wrongly recognized as idle state or left/right hand motor imagery. Here, the F-measure is used for evaluating the performance of motor imagery detection [20,21], and the value of the F-measure can be expressed by:

(3) $$F\_measure=2\times \frac{precision\times recall}{precision+recall}\times 100\%$$

where precision and recall denote positive predictive value (PPV) and sensitivity respectively, and can be calculated by:

(4) $$precision=\frac{tp}{tp+fp}\times 100\%$$

(5) $$recall=\frac{tp}{tp+fn}\times 100\%$$

**Figure 9:**
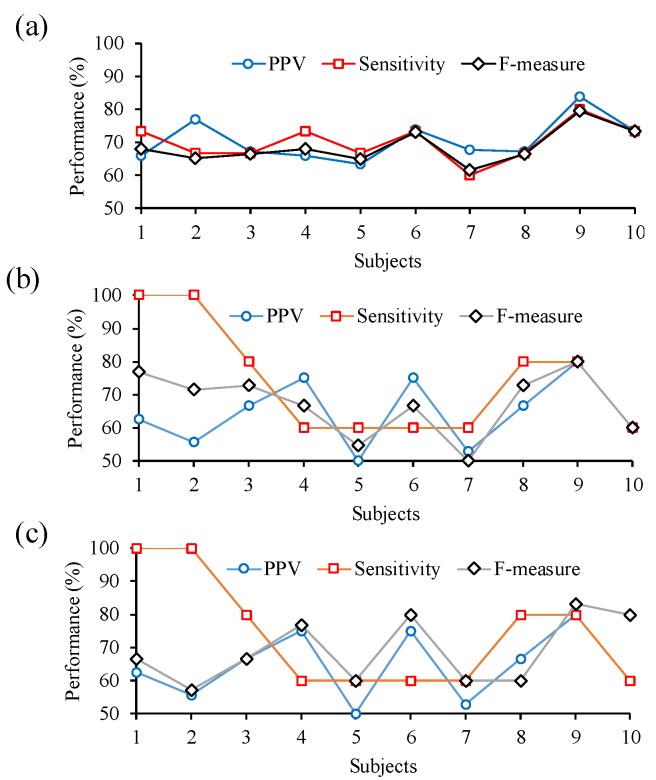
Experimental results for PPV, sensitivity, and F-measure of detecting (**a**) all motor imagery; (**b**) right hand motor imagery; and (**c**) left hand motor imagery.

Figure 9a–c show the PPV, the sensitivity, and the value of F-measure for all, right hand and left hand motor imagery detection respectively. The averaged PPV, sensitivity, and F-measure for motor imagery detection are 70.5% ± 6.3%, 70% ± 5.7%, and 68.6% ± 5.2%, respectively. The accuracy for motor imagery detection is about 71.1% ± 5%.

Next, the information transfer rate (ITR) is used for evaluating the performance of the proposed BCI system [22,23], and it can be calculated by:

(6) $$B=\log_{2}\left(N\right)+P\log_{2}\left(P\right)+(1-P)\log_{2}[(1-P)/(N-1)]$$

where the parameters *B*, *N*, and *P* denote the information transfer rate (bits/trial), the number of mental events, and the accuracy. The proposed system could detect three mental events, including the right and the left hand motor imagery, and the idle state. Therefore, the information transfer rate of the proposed system is about 3.2 bits/min.

## 4. Discussion

The g.tec Company (city, state abbrev if USA, country) has also proposed an active dry electrode (g.SAHARA) for the EEG measurement [24]. Here, the metal electrode part was connected with an active electrode circuit. In general, a capacitor was usually series connected to the input node of the operational amplifier of the active circuit to eliminate the DC offset, and this might contribute to the effect of phase distortion [25]. Moreover, the pin length of metal electrode part was constant. Therefore, it might contact with the skin incompletely, in particular, under motion. In this study, the retractable comb-shaped dry active electrode is designed to measure EEG signals on hairy sites without conductive gel. The comb-shaped structure could separate the hair layer, and the retractable spring loaded pins could provide a suitable pressure to maintain a good skin-electrode contact condition. Moreover, the metal electrode part directly connected with the active circuit of the proposed electrode could provide an ultra-high input impedance and ultra-low input current to reduce the influence of high skin-electrode interface impedance. From the experimental results, the correlation between the EEG signals obtained by the proposed electrodes and the wet electrodes is high, and their coherence is also high. Therefore, compared with the EEG signals obtained by wet electrodes, the phase distortion of the proposed electrodes corresponding to difference frequencies is very slight. The proposed retractable comb-shaped dry active electrodes could successfully measure the EEG signals in hairy site, without conductive gel, and could provide a good EEG signal quality similar to that by the wet electrodes. By the design of the proposed electrodes, it effectively improves the convenience of use in daily life.

The experimental channel selection results show that the locations of the selected channels corresponding to the right and the left hand motor imagery are most frequently at C3 and C4, respectively, and the motor imagery event could exactly be reflected on the ERD quantifications at C3 and C4. The brain areas around C4 and C3 correspond to the primary motor cortex areas, which are mainly responsible for the movement of limbs, hands and fingers. A previous study [26] indicated that the change of ERD features associated with hand motion and motor imagery could be responded to at the locations C3 and C4. Therefore, the experimental results fit the phenomenon mentioned in the previous study. The locations of the selected channels might also be at F3, F4, P3, and P4 due to individual differences [13].

Several motor imagery-based BCIs have been proposed in previous studies, and a comparison between the proposed BCI and other BCI systems is presented in Table 1. Pfurtscheller *et al.*, proposed a Graz BCI, and the approaches of linear discrimination analysis and neural networks were used for the EEG feature classification. The accuracy of classifying the left and the right hand motor imagery was about 65% [27]. Lan *et al.*, proposed an offline channel selection approach with the technique of mutual information, which was used for ranking all of the channels and then selecting them. Here, 32 EEG channels using wet electrodes were used for acquiring EEG signals, and several related-feature channels were selected and used for classifying two mental tasks of motor imagery. The accuracy of mutual information-based channel selection was around 80% [12]. Arvaneh *et al.*, proposed the sparse common spatial pattern (SCSP) algorithm to remove noisy or irrelevant channels as a channel selection. Here, 22 channels and 118 channels from different datasets were used, and about 13 channels and 22 channels were selected for motor imagery detection, respectively. The average accuracy of detecting two motor imagery tasks by using 22 and 118 EEG channels was about 81% and 82% [13]. Kus *et al.*, proposed a multi-class motor imagery-based BCI, based on a multinomial logistic regression classifier, to detect the right hand, the left hand and the feet imagery. The accuracy was 74.8% and the information transfer rate with three mental tasks was 4.5 bits/min [28]. Obermaier *et al.*, proposed a five-class brain-computer interface with the technique of EEG patterns to detect 2~5 mental tasks, and the information transfer rate with two mental tasks was about 3.15 bits/min [29]. Most of above BCI systems acquired EEG signals by using wet EEG electrodes and the EEG machine and performed BCI algorithm in the back-end computer. Moreover, most of above BCI systems were not wearable systems. The above issues increased the limitation of BCI applications.

**Table 1:** Comparison between other BCI systems and the proposed BCI.

	Lan *et al.* [12]	Arvaneh *et al.* [13]	Pfurtscheller *et al.* [27]	Kus *et al.* [28]	Obermaier *et al.* [29]	Proposed BCI
Accuracy (%)	80	81/82	65	74.8	-	71.1
Bit rate(bits/min)	-	-	-	4.5	3.1	3.2
EEG features	Power spectral density	Common spatial pattern	Band power estimation	Spectral power estimation	EEG Pattern	EEG Power
Number of EEG channels	32	22/118	32	-	29	8
Function of channel selection	Yes	Yes	No	No	No	Yes
EEG sensor	EEG cup electrode	EEG cup electrode	EEG cup electrode	EEG cup electrode	EEG cup electrode	Noevl dry electrode
Main computing unit	Back-end computer	Back-end computer	Back-end computer	Back-end computer	Back-end computer	Front-end wearable EEG device
Wearable system	No	No	No	No	No	Yes
Wireless transmission	WiFi	No	No	No	No	Bluetooth

The average accuracy of detecting motor imagery and ITR for the proposed system is about 71.1% ± 5%, and 3.2 bits/min, respectively. Therefore, the performance of the proposed system for detecting motor imagery with only eight EEG channels is similar to that of the above BCI systems. In order to maintain stable performance of EEG feature detection related to motor imagery, three consecutive motor imagery events have to be performed in each testing cycle, and this also slightly reduces the ITR performance. However, different from the above BCI systems, the proposed system acquires EEG signals and performs BCI algorithm in a front-end wearable EEG device. This also greatly improves the convenience of use.

## 5. Conclusions

In this study, a novel wearable channel selection-based brain-computer interface for motor imagery detection was proposed. By using retractable comb-shaped dry active electrodes and a wearable mechanical design, it could easily measure a good EEG signal quality from a hairy site without conductive gel and effectively improved the convenience of use in daily life. From the experimental results, the proposed electrodes could precisely provide a good EEG signal quality similar to those wet electrodes provide. In the front-end wearable EEG device, an analog CAR spatial filter circuit was designed to perform the function of spatial filter without any calculation. From the experimental results, the ERD feature corresponding to the right/left hand motor imagery could easily be extracted. Moreover, by building the channel selection function into the firmware of the wireless EEG acquisition module, the channel selection function could be performed in the front-end device, and only EEG signals of the selected EEG channels were transmitted to the back-end host system to effectively reduce the data transmission load and easily integrate this design with other commercial electronic devices, such as tablets and mobile phones, to reduce the limitations of BCI applications. Therefore, the proposed system can be viewed as a good system prototype of wearable BCI systems.
